# Alternative Cancer Therapeutics: Unpatentable Compounds and Their Potential in Oncology

**DOI:** 10.3390/pharmaceutics16091237

**Published:** 2024-09-23

**Authors:** Dmitriy Ovcharenko, Dmitry Mukhin, Galina Ovcharenko

**Affiliations:** Altogen Labs, 11200 Menchaca Road, Austin, TX 78748, USA

**Keywords:** cancer, alternative therapeutics, off-label drugs, regulatory challenges, natural remedies, combination cancer therapy

## Abstract

Cancer remains a leading cause of death globally. Cancer patients often seek alternative therapies in addition to, or instead of, conventional treatments like chemotherapy, radiation, and surgery. The progress in medical advancements and early detection provides more treatment options; however, the development of cancer drugs requires a significant amount of time, demands substantial investments, and results in an overall low percent of regulatory approval. The complex relationship between patent protection and pharmaceutical innovation complicates cancer drug development and contributes to high mortality rates. Adjusting patent criteria for alternative cancer therapeutics could stimulate innovation, enhance treatment options, and ultimately improve outcomes for cancer patients. This article explores the potential of alternative cancer therapeutics, chemopreventive agents, natural products, off-patent drugs, generic unpatentable chemicals, and repurposed drugs in cancer treatment, emphasizing the mechanisms and therapeutic potential of these unconventional compounds as combinatorial cancer therapies. The biological pathways, therapeutic effects, and potential to enhance existing therapies are reviewed, demonstrating their cost-effective and accessible options as adjuvant cancer therapies.

## 1. Introduction

This study aims to highlight the potential of alternative cancer therapies by examining their biological mechanisms, therapeutic effects, and abilities to enhance existing treatments. While numerous alternative therapies have been documented, their efficacy and safety often remain unverified due to limited published research. Expanding preclinical studies on these compounds is crucial for broadening access to safe cancer therapies and advancing oncological research. The importance of existing alternative cancer therapies is emphasized, alongside the value of collaborative models, including international partnerships and AI-driven drug discovery. Such collaborations are essential for addressing funding gaps and accelerating the development of unconventional therapies. Overcoming regulatory and financial challenges is critical to uncovering cost-effective and accessible cancer treatments that complement existing therapies and improve patient outcomes.

**Figure 1:**
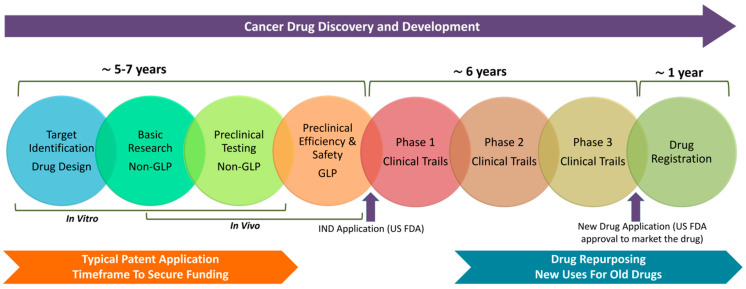
Main steps in cancer drug discovery and development process. Types of experimental activity, regulatory steps, and associated timeline estimates are schematically represented.

The cancer drug discovery and development process in the United States is regulated by the U.S. Food and Drug Administration (US FDA) and typically takes about 12–14 years (Figure 1). It begins with discovery and preclinical testing to identify and assess potential drug targets. An Investigational New Drug (IND) application must be approved by the US FDA before clinical trials can commence. Clinical trials occur in three phases: Phase I (safety and dosage), Phase II (efficacy and side effects), and Phase III (confirmation of effectiveness and comparison with existing treatments). Only about 25–30% of drugs make it through these phases to earn US FDA approval. After clinical trials, a New Drug Application (NDA) is submitted for US FDA review. Approved drugs enter post-market surveillance to monitor any long-term or rare side effects. Some studies show that the overall probability of a new cancer drug reaching US FDA approval is about 3–5% [1,2].

The development of innovative cancer treatments entails a comprehensive process, which includes drug discovery, preclinical testing for efficacy and safety, and the acquisition of regulatory approval. Patent protection plays a crucial role in recouping these investments; however, the efficacy of patents is contingent upon adherence to intricate legal nuances, with any lapses potentially diminishing their value significantly. A significant challenge within the pharmaceutical industry is the non-patentability of many chemical compounds that are part of the public domain. The current intellectual property (IP) framework suggests that without patent protection, the dissemination of medical and life sciences research could become unrestricted, complicating the translation of these advancements into patient benefits. In many jurisdictions, manufacturers of non-patentable substances, like dietary or food supplements, are not required to prove safety or efficacy through clinical trials as long as no therapeutic claims are made. They must still comply with general safety regulations and truthful labeling practices. This regulatory gap often leads to the promotion of such products without adequate evidence of their safety or therapeutic value, posing risks to public health and undermining trust in the pharmaceutical sector [3,4]. The absence of patentability removes the financial incentive for research and development into the safety and efficacy of these compounds, leaving their potential therapeutic benefits largely unexplored. Furthermore, the frequent violation of policies regarding therapeutic claims exacerbates these issues, highlighting the need for a more rigorous and formalized approach to pharmaceutical development and patent law to ensure the advancement of safe and effective cancer treatments [5,6].

Off-patent drugs and non-oncology compounds are often investigated for other cancer indications and can be used for drug repurposing [7,8], as they have an established safety profile. However, many unconventional compounds with demonstrated anti-cancer biological activity cannot be transferred into clinical practice, as they were never tested in clinical trials, and have no preclinical safety data established. Evaluating the efficacy and safety of these chemicals, including their analogs and derivatives, could broaden the access to therapeutic molecules, advancing oncological research. This approach leverages existing drugs for new uses, facilitating quick clinical translation and a multi-target strategy against cancer. The review of the mechanisms of action of existing unpatentable chemicals with known anticancer activity provides insights into how these compounds can be effectively utilized in cancer treatment. By exploring the biological pathways and therapeutic impacts of these chemicals, this paper underscores their potential in augmenting existing treatment modalities, highlighting the necessity for more inclusive research approaches that embrace both patented and non-patentable solutions for combating cancer.

## 2. Patenting of Anticancer Compounds

The USA is considered a leading country in regards to providing a superior intellectual property environment [9,10] and the U.S. Patent Act delineates the criteria that must be satisfied for a drug to be eligible for patent protection. Given the substantial costs associated with meeting approval requirements and the risk posed by competitive pressures, pharmaceutical companies tend to be selective in the cancer treatments they choose to develop. Consequently, promising treatments often never reach commercialization [11,12].

Patents incentivize innovation by providing legal protection, but they require that inventions meet specific stringent criteria. Drugs that fail to meet these criteria are non-patentable, and judicial exceptions include natural products, abstract ideas, and natural phenomena. Generic drugs, deemed bioequivalent to branded drugs by the US FDA, may be marketed once the patent exclusivity expires. The high costs of clinical trials and the potential for generics to gain approval using this data discourage companies from developing any type of non-patentable drugs, which may result in effective cancer treatments remaining unavailable to the public. Approximately 50–60% of U.S. cancer patients use complementary and alternative medicines, either alone or alongside conventional treatments [13,14]. These substances present potential benefits and challenges in cancer treatment.

The patent system, designed to incentivize innovation in pharmaceuticals and medical devices, has faced criticism for increasing healthcare costs and hindering research by limiting access to patented materials [6,15]. A notable example: The U.S. Orphan Drug Act, which has facilitated the introduction of innovative treatments for rare cancers, some of which have found applications in treating other, more common diseases. The field of oncology has emerged as a significant contributor to orphan drug research, as evidenced by the fact that over one-third of all orphan drugs approved by the U.S. Food and Drug Administration (US FDA) are intended for oncological use [16,17].

Many anticancer compounds face challenges in patentability for several reasons, often rooted in the nature of the compounds themselves, the legal requirements for patentability, and the complex landscape of cancer treatment research. The eligibility for patent protection depends on fulfilling the criteria of novelty, invention, utility, and applicability, which many anticancer compounds don’t meet. Most natural products are not patentable because they are found in nature, whereas only inventions (not discoveries) are eligible for patents. This poses a significant hurdle, as many natural compounds are identified rather than created. A major challenge in patenting natural products and previously known compounds is demonstrating the novelty and non-obviousness required for patent eligibility [18]. To qualify under the criteria for novelty, the compound as an anticancer agent must be unprecedented in any public disclosure, encompassing all prior publications, or commercial activities. The applicability also necessitates a demonstration of the compound’s practical therapeutic utility in cancer treatment, substantiated by empirical evidence such as preclinical or clinical efficacy data.

Numerous compounds and chemicals previously documented in scientific literature are now recognized for their anticancer properties, demonstrating potential for their therapeutic applications; however these compounds are considered unpatentable [19]. While the compounds directly extracted from nature may not be patentable, processes used to isolate, purify, or chemically modify these compounds can be. This approach often requires significant investment in research and development to create a patentable product. Given the significant hurdles involved in directly patenting many anticancer compounds, researchers and pharmaceutical companies have developed nuanced strategies to navigate the patent landscape effectively (Table 1).

**Table 1:** Common strategies for meeting the stringent requirements of patentability criteria for anticancer compounds.

Novel Formulations	Developing new formulations of existing drugs can offer ways to improve their efficacy, reduce side effects, or enhance delivery to the target site. For instance, encapsulating a drug in a nanoparticle might improve its solubility or allow it to target cancer cells more effectively. Innovative formulations can be patentable, as they present new and non-obvious solutions [20,21].
Drug Delivery Systems	Similar to novel formulations, advancements in drug delivery systems offer significant opportunities for patentability. Targeted delivery mechanisms, time-release capsules, and transdermal patches that improve the drug’s performance or patient experience can be patented. Such systems can transform how a drug is administered and delivered, making a substantial difference in treatment outcomes [22,23].
Synthetic Derivatives	Even if a natural compound itself cannot be patented, chemically modified derivatives that show improved properties (higher potency or lower toxicity) may be. Researchers often focus on altering the molecular structure of known compounds to create new, patentable entities that retain or enhance the desired anticancer activity [24,25].
Combination Therapies	Patenting the use of known drugs in combination can be another avenue for innovation. If two or more drugs are found to work synergistically, where their combined effect is greater than the sum of their individual effects, this combination can be patented. This approach opens new therapeutic avenues and extends the commercial life of existing drugs [26,27,28].
Methods of Use	Even when the compounds themselves are not new, novel applications or methods of using them can be patentable. Discovering and proving a new use for an existing drug, such as using a known medication in treating a different type of cancer than it was originally approved for, can lead to patent protection for that specific application [29,30].
Production Processes	Innovations in the methods for manufacturing or synthesizing anticancer compounds can also be protected by patents. Efficient, scalable, and environmentally friendly production methods that are novel and non-obvious offer significant competitive advantages and are valuable in the patent landscape [31,32].

These strategies (Table 1) illustrate how the biomedical field continues to evolve in its approach to patenting, reflecting a broader understanding that innovation can take many forms. By focusing on these areas, researchers and companies not only navigate around the challenges of patenting anticancer compounds but also contribute to the advancement of cancer treatment, ensuring that new and improved therapies continue to reach patients.

## 3. Alternative Cancer Therapeutics: Combinatorial Approach and Patentability

The use of alternative and unconventional therapies in cancer treatment has historical roots, originating from cultural traditions and holistic practices dating long before the birth of modern scientific medicine. Despite the dominance of pharmaceuticals and surgery in the 19th and 20th centuries, alternative therapies have persisted as complementary options. These approaches provide patients with a broader range of treatment strategies, enhancing recovery and well-being, as well as reducing the risk of recurrence. The limitations of conventional treatments, particularly in managing cancers in which the mortality rates remain high, have driven the need for complementary alternatives. Combination therapies have proven more effective than monotherapy by employing drugs with different mechanisms of action, minimizing the likelihood of drug resistance. These regimens, which include immunotherapy, chemotherapy, and radiotherapy, address the complexity of cancer and aim to prevent resistance by targeting diverse molecular pathways. As cancer cells adapt and evolve, resistance to treatment becomes a major challenge, necessitating innovative approaches to therapy. By combining agents that act on distinct cancer growth mechanisms, combination therapies offer a comprehensive strategy to manage the disease’s complexity and improve therapeutic outcomes.

Cancer’s heterogeneity at the genetic, epigenetic, and proteomic levels makes treatment particularly challenging. Combination therapies, which target multiple aspects of tumor biology, provide a more nuanced approach, enhancing treatment response and prolonging remission. While monotherapy remains beneficial for some, the simultaneous targeting of multiple pathways through combination treatments offers greater potential to hinder tumor resistance and achieve durable responses. However, the reliance on synergistic mechanisms, in which the combined effects of two agents exceed their individual efficacy, can introduce challenges, including unwanted side effects and vulnerabilities to resistance. To address these challenges, advanced preclinical model systems such as patient-derived xenografts and organoids are being used to better represent tumor complexity and refine combination strategies. Additionally, advances in artificial intelligence are expected to accelerate the discovery of more effective combinations.

While alternative cancer therapeutics may not be patentable as standalone treatments, the combination of conventional cancer drugs with alternative cancer therapeutics can be patented, under specific conditions (Figure 1). Even if the individual components are well-established and widely known, a unique combination that has not been previously disclosed or documented may satisfy the criteria for novelty and non-obviousness. Such combinations can demonstrate synergistic or unexpected effects, such as enhanced therapeutic efficacy, reduced side effects, or improved patient outcomes, which contribute to their patentability. To qualify for a patent, the combination therapy must demonstrate clear therapeutic utility, such as improving the effectiveness of cancer treatment, minimizing adverse reactions, or addressing the challenge of drug resistance. A new formulation that incorporates both conventional cancer drugs and alternative therapeutics, and that achieves these benefits, could be considered patentable. The patent claims for such a combination could focus on various aspects, including the specific method of treatment, the formulation of the therapy, or the synergistic interaction between the components. These claims must clearly describe how the combination operates to provide an improved therapeutic outcome, which may distinguish it from previously known treatments. This approach underscores the potential for innovative therapies that leverages both conventional and alternative methods in cancer treatment to gain intellectual property protection. The combination therapies represent a significant potential in cancer treatment, offering a more comprehensive approach to tackling the disease’s complexity and resistance. Additional patenting strategies can be discussed to accelerate the adoption of alternative cancer therapeutics into clinical applications (Table 2). As technologies continue to evolve, the development of combination strategies will further enhance patient outcomes and reduce cancer-related mortality.

## 4. Patient Perspectives and Ethical Considerations in Alternative Cancer Therapeutics

Cancer patients often seek alternative therapies as complementary or substitute options to conventional treatments like chemotherapy, surgery, and radiation. These alternative treatments may be appealing due to their perceived lower toxicity, potential cost savings, and alignment with personal beliefs or cultural practices. However, the efficacy and safety of such alternatives are frequently unverified by rigorous clinical trials or even the existence of preclinical data, creating significant uncertainty in regards to patient decision making [33]. The accessibility and affordability of alternative therapies are critical concerns, exacerbating disparities associated with healthcare access. Ethical concerns in alternative cancer therapeutics primarily revolve around informed consent and patient autonomy. Healthcare providers are ethically obligated to ensure that patients are fully informed of the risks and benefits of alternative treatments, with their advice being grounded in the best available evidence. Balancing respect for patient autonomy with the duty to recommend evidence-based treatments presents a significant ethical challenge, particularly when patients choose less proven alternatives. Additionally, the promotion of unproven therapies raises ethical questions about potential exploitation and health risks [34].

The patenting of cancer therapeutics significantly influences patient access. Patents, while encouraging innovation by protecting intellectual property, can result in high drug prices, restricting access to life-saving treatments for many patients, particularly in low- and middle-income regions. This raises ethical concerns about the fairness of a healthcare system that allows economic barriers to limit access to essential medications [35]. Moreover, the patenting of natural substances used in alternative therapies can further restrict access by transforming affordable treatments into costly patented drugs, thereby limiting patient access to previously accessible options. The ethical challenges regarding alternative cancer therapeutics, patient perspectives, and the impact of patent laws on access to medications are interrelated. Addressing these challenges requires a focus on transparency, informed consent, and re-evaluating the role of patent laws to ensure equitable access to effective cancer treatments for all cancer patients.

## 5. Cancer Chemopreventive Agents

Most of research studies indicate that a single genetic alteration is insufficient to drive cancer, as alternative mechanisms often lead to genomic repair or cell death. A lethal metastatic property requires extensive genetic and epigenetic modifications, enabling uncontrolled proliferation, invasion, and metastasis, particularly in the elderly population. Despite advances in understanding the etiology and molecular mechanisms of cancer, these have not significantly improved outcomes for patients with late-stage cancers. Additionally, chemotherapeutic agents often cause severe side effects, reducing patients’ quality of life. Therefore, strategies to reduce cancer incidence or prevent the progression of benign neoplasms to advanced cancers are crucial.

Cancer chemoprevention involves using synthetic or natural bioactive agents to suppress, prevent, or delay tumorigenesis by blocking the initiation stage of carcinogenesis or inhibiting the promotion stage, where initiated cells proliferate into a tumor [36,37]. The concept, first introduced in the 1970s, has evolved with the identification of numerous agents capable of preventing or delaying the onset of cancer due to their potential to halt carcinogenesis at various stages. These agents are classified into dietary compounds, pharmaceuticals, and natural products, each offering distinct mechanisms of action. Many preclinical studies have demonstrated the efficacy of phytochemicals and dietary components as chemopreventive agents [38,39,40].

As with chemotherapeutic drug development, testing potential chemopreventive agents involves multiple phases of human studies. Numerous clinical trials have assessed the chemopreventive effectiveness of compounds based on epidemiological or preclinical data. Chemopreventive agents have shown a success in protecting high-risk populations from cancer, suggesting chemoprevention as a promising strategy [36,37]. However, some clinical studies have shown that certain agents, instead of preventing cancer, either had no effect or, in some cases, increased cancer incidence, effectively turning these agents into carcinogens rather than chemopreventive agents [41,42,43]. This underscores the critical importance of conducting thorough preclinical research and well-designed clinical studies before any compound is used in humans as a cancer therapy. Rigorous testing in the preclinical phase is essential to identify potential risks, while precise and carefully controlled clinical trials are necessary to ensure the safety and effectiveness of these agents.

The α-tocopherol and β-carotene clinical trial found higher lung cancer rates with the used of β-carotene. A placebo-controlled clinical trial study including 29,133 men showed that those on β-carotene experienced an 18% increase in lung cancer and cardiovascular disease incidence and an 8% rise in overall mortality [41]. The β-carotene and retinol efficacy trial, involving individuals with asbestos exposure or a history of smoking, was halted due to higher lung cancer and cardiovascular disease mortality in the intervention group [42] and reported a 28% increase in lung cancer incidence. Additionally, a study on NSAIDs indicated an increased risk of small cell lung cancer in those taking regular-strength aspirin [43].

Compounds that block the initiation stage are termed blocking agents, while those affecting the promotion stage are suppressing agents [36]. Blocking agents work by reducing the metabolic activation of pro-carcinogens into carcinogens, decreasing reactive oxygen species (ROS) levels, and inducing genomic repair pathways [44,45]. They may also prevent tumors by modulating epigenetic modifications, such as the hypermethylation of tumor suppressor genes [46,47]. Suppressing agents exert their effects by inhibiting signaling pathways that promote cell survival and proliferation (Table 3).

**Table 3:** Selected chemopreventive phytochemicals: mechanisms of action and sources.

Compound	Stage Affected	Mechanism of Action	Source
Curcumin	Initiation and Promotion	Downregulates multiple survival signals; inhibits transcription factors, including NF-κB.	*Curcuma longa*, turmeric plant [48,49]
Epigallocatechin-3 Gallate	Initiation	Reduces tumor invasiveness; sensitizes cells to other treatments like tamoxifen.	Prevalent in green tea [50,51]
Resveratrol	Initiation and Promotion	Causes G1/S phase arrest, downregulates COX-1 and COX-2, and inhibits multiple signaling pathways.	Grapes, fruits, nuts [52,53]
Tryptanthrin	Promotion	Suppresses PMA-induced proliferation; downregulates pro-tumorigenic signaling pathways.	*Strobilanthes cusia*, other medicinal plants [54,55]
Kaempferol	Initiation and Promotion	Inhibits growth of cancer cells and induces autophagic cell death via signaling pathways.	Abundant in vegetables and medicinal herbs [56,57]
6-Gingerol	Initiation and Promotion	Induces apoptosis, inhibits MAPK/AP-1 signaling, and scavenges chemical carcinogens.	*Zingiber officinale*, ginger plant [58,59,60]
Emodin	Initiation	Inhibits AP-1 and NF-κB signaling pathways; inhibits angiogenesis and metastasis.	*Rheum palmatum*, *Polygonum cuspidatum*, and other plants [61,62,63]

## 6. Therapeutic Potential of Unpatentable Compounds in Cancer Treatment

**Figure 2:**
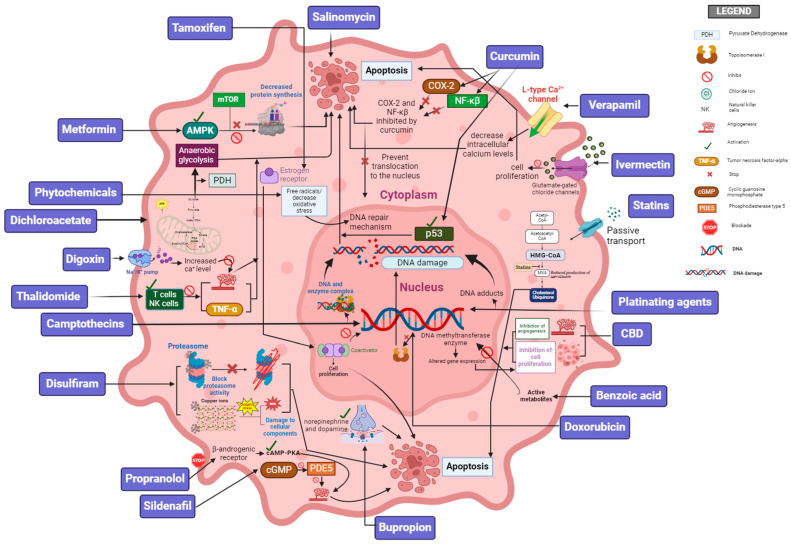
Anticancer mechanism of action of selected adjuvant chemotherapy compounds.

The field of cancer therapy is evolving, with a significant shift from traditional treatments like chemotherapy, radiation therapy, and surgery to innovative approaches targeting specific molecular pathways in tumorigenesis. Unpatentable chemical compounds and off-patent drugs often serve as cost-effective alternatives to branded medications. These compounds offer a diverse range of mechanisms of action, making them promising for combination therapies, personalized and targeted treatments, and overcoming drug resistance in cancer. Their accessibility and potential efficacy position them as valuable tools for developing new strategies to improve patient outcomes in oncology. This exploration into non-patentable and off-patent compounds represents an important direction in cancer research, promising to enhance oncology treatment efficacy, overcome challenges like drug resistance, and pave the way for more effective, personalized cancer care. Despite the challenges accompanying unpatentable compounds, such as the lack of financial incentives and the existence of regulatory hurdles, there are important benefits in terms of cost, accessibility, and potential efficacy that underscore the importance of continued research and development (Figure 2).

### 6.1. Metals and Minerals

Zinc plays a critical role in the structural stabilization, DNA repair, and activation of p53, a key protein in the apoptotic process, as well as in the activation of caspases. By modulating these enzymes, zinc promotes cancer cell death. Zinc enhances the antioxidant capacity of the cell, protecting normal cells from oxidative damage while inducing apoptosis in cancer cells through ROS-mediated pathways. It has been shown to have beneficial effects against chemically induced preneoplastic progression in rats, suggesting its potential as a dietary chemopreventive agent for individuals at high risk of cancer. Research on both animals and humans indicates that zinc deficiency is a significant factor in cancer development and progression. Zinc supplementation could therefore be effective in preventing and treating various cancers, including those of the colon, pancreas, esophagus, and head and neck [64].

Arsenic has been studied extensively for its anti-cancer properties. It interferes with the cell cycle by affecting the activity of cyclins and cyclin-dependent kinases, which are crucial for cell cycle progression and which can also induce apoptosis. This disruption halts the proliferation of cancer cells. The generation of reactive oxygen species (ROS) by arsenic leads to oxidative stress, causing damage to cellular components and further promoting apoptosis. Arsenic itself is a poison, causing health issues affecting millions due to environmental and occupational exposure [65]. While arsenic is highly toxic in its inorganic form, synthesized arsenic nanoparticles show potential for cancer treatment [66].

Gold compounds exhibit anti-cancer effects through several mechanisms. They inhibit crucial enzymes like thioredoxin reductase, essential for maintaining cellular redox balance. The inhibition of this enzyme results in increased ROS levels, which induce oxidative stress and damage cellular components, leading to apoptosis [67]. Gold compounds also interact with DNA and RNA, disrupting their normal functions and hindering cancer cell replication. Moreover, they interfere with key cellular signaling pathways such as PI3K/AKT and MAPK/ERK, which are vital for cancer cell survival and proliferation. Gold compounds can inhibit angiogenesis by downregulating factors like VEGF, preventing the formation of new blood vessels necessary for tumor growth and metastasis [68].

Selenium has demonstrated significant anti-cancer properties by inducing oxidative stress in cancer cells, leading to DNA and cellular damage that triggers apoptosis. At lower concentrations, selenium exhibits anti-inflammatory properties and enhances the body’s antioxidant defenses, protecting normal cells from oxidative damage. Selenium compounds have been shown to modulate various cell signaling pathways, including p53 tumor suppressor, Bax/BCL-2 apoptosis regulation, and nuclear factor-kappa B (NF-κB) cell survival, promoting cancer cell death and inhibiting processes critical for cancer cell growth, migration, and invasion [69]. Dietary selenium, through its incorporation into selenoproteins, regulates inflammation and immunity by supporting immune cell activation, differentiation, and proliferation, while preventing excessive immune responses and chronic inflammation. Selenium deficiency can impair immune functions due to increased oxidative stress and other cellular dysfunctions [70].

Copper plays a role in cancer treatment by reducing angiogenesis. Copper chelators inhibit factors like VEGF and fibroblast growth factor (FGF), which are essential for tumor vascularization. Additionally, copper ions can generate ROS, leading to oxidative stress and apoptosis in cancer cells [71]. Organometallics like copper compounds are used in cancer chemotherapy, either alone or with other drugs. Certain copper complexes effectively inhibit topoisomerases, enzymes involved in DNA topology regulation. These inhibitors work through various mechanisms, including promoting DNA breaks, acting catalytically, intercalating with DNA, and generating reactive oxygen species (ROS). These actions impact cell cycle checkpoints and death effectors. The study of copper complexes as topoisomerase inhibitors includes their synthetic development and their effects on cancer cell mutations. Emerging treatment aspects highlight the potential for expanding the use of these potent anticancer agents in clinical trials and future therapies [72].

For over four decades platinum-based chemotherapeutic agents, notably cisplatin, carboplatin, and oxaliplatin, have occupied a central role in the oncological pharmacopeia, serving as cornerstone treatments across a broad spectrum of malignancies. These compounds have garnered widespread acclaim within the medical community for their efficacy in cancer management, frequently being designated as the primary modality of treatment, despite their association with significant adverse effects, pronounced systemic toxicity, and the emergence of cellular resistance. The mechanism of action of platinum-based drugs is intricately linked to their capacity to induce platinum–DNA crosslinks within cancer cells. This interaction disrupts the integrity of the DNA structure, precipitating a cascade of molecular events that culminate in cell death. Concurrently, these drugs activate a variety of signal transduction pathways, further augmenting their antineoplastic activity. Despite their potent anticancer properties, the clinical utility of platinates is impeded by the development of resistance mechanisms. These include diminished intracellular drug accumulation, detoxification by cellular antioxidants, and the activation of DNA repair mechanisms, all of which serve to mitigate the cytotoxic effects of these agents [73]. Contemporary research endeavors are increasingly focused on elucidating the molecular underpinnings of resistance to platinum-based therapies. This includes the identification of genetic determinants that may predispose resistance, alongside a detailed exploration of the adverse effects engendered by these treatments. In parallel, there is a significant interest in the integration of platinum-based drugs within the paradigm of targeted cancer therapy. This approach seeks to leverage the cytotoxic potential of platinates, employing them as active components within carrier molecules designed to deliver therapeutic agents directly to tumor cells. The advent of nanotechnology has been particularly transformative in this context, facilitating the development of platinum-based nanodrugs [74]. These innovative formulations promise to enhance the specificity and efficacy of cancer therapy, potentially circumventing the limitations imposed by systemic toxicity and drug resistance.

### 6.2. Organic Compounds

The anticancer properties of salicylic acid (SA) and acetylsalicylic acid have been described to induce the pro-apoptotic effects in cancer cells. The mechanism of action involves the induction of endoplasmic reticulum (ER) stress, leading to the activation of apoptotic pathways. Studies have shown that treatment with salicylic acid triggers a sequence of molecular events, beginning with the activation of nitric oxide synthase 3 (eNOS) through an AKT/mTOR/AMPK-dependent pathway. This activation results in increased production of nitric oxide and reactive oxygen species, which are critical mediators of the ER stress response. The culmination of this stress response is the upregulation of the pro-apoptotic transcription factor C/EBP homologous protein (CHOP), marking a critical step in the initiation of apoptosis in cancerous cells [75]. These findings highlight the complex molecular mechanism of salicylic acid treatment, providing insight into how this widely used compound might be repurposed to target and eliminate cancer cells through the induction of apoptosis. The potential therapeutic implications of these results suggest that the use of salicylic acid-based compounds with well-established safety profiles could be integrated into existing cancer treatment regimens to enhance their efficacy. Further research in this area is warranted to fully understand the mechanisms of action and to optimize the therapeutic application of salicylic acid-based compounds in oncology. Salicylic acid has been observed to reduce the risk of colorectal cancer. Acetylsalicylic acid, which quickly deacetylates to SA, serves as an effective agent for both primary and secondary chemoprevention [76].

Statins, primarily known for their lipid-lowering effects, have emerged as potential anticancer agents, revealing a complex interplay between lipid metabolism and cancer development. Their mechanism of action involves inhibiting the 3-hydroxy-3-methylglutaryl-coenzyme A (HMG-CoA) reductase enzyme, pivotal in the mevalonate pathway, which is crucial for the synthesis of cholesterol and non-sterol isoprenoids. This inhibition leads to reduced cholesterol levels and impacts several cancer-related pathways, including inhibition of RAS and Rho isoprenylation, signal transduction, and DNA synthesis. Statins have been observed to exhibit pleiotropic effects, influencing various aspects of cancer biology including cell proliferation, angiogenesis, metastasis, as well as inducing cell cycle arrest, oxidative stress, autophagy, and apoptosis in cancer cells [77]. The regulation of autophagy plays an important role in the tumor suppressive process, especially during the early tumor development stages. Several studies demonstrated that statins can induce ferroptosis and pyroptosis. Clinical and epidemiological studies have shown an association between statin use and a decreased risk of cancer incidence, improved survival rates in cancer patients, and potential synergistic effects when used in combination with other chemotherapeutic agents. The clinical application of statins in oncology requires further investigation to fully understand their mechanism of action, optimal dosing, and potential therapeutic benefits in cancer prevention and treatment [78].

### 6.3. Natural Products

Curcumin, a polyphenolic compound derived from the rhizome of the turmeric plant (*Curcuma longa*), was demonstrated to exhibit anti-cancer activity through several distinct mechanisms, including the modulation of several cell signaling pathways that are critical for cell growth and survival, including the NF-κB (nuclear factor kappa-light-chain-enhancer of activated B cells), Wnt/β-catenin, and PI3K/AKT pathways. By downregulating the activity of these pathways, curcumin inhibits the proliferation of cancer cells and induces apoptosis, thereby limiting tumor growth and promoting tumor regression. The anti-inflammatory effects resulting from the suppression of the production of pro-inflammatory cytokines and enzymes (TNF-α and COX-2) contribute to its anticarcinogenic properties. Antioxidant activity was also described; by scavenging reactive oxygen species (ROS) and enhancing the expression of antioxidant enzymes, curcumin can protect cells from DNA damage and mutagenesis, which are pivotal events in the carcinogenesis process. Curcumin has been shown to modulate microRNA expression, influencing various biological processes, including inflammation, cancer, and cardiovascular diseases, by regulating gene expression at the post-transcriptional level [79,80]. It also plays a role in the modulation of the tumor microenvironment and angiogenesis by inhibiting the expression of various angiogenic factors, such as vascular endothelial growth factor (VEGF), thereby disrupting the blood supply required for tumor growth and metastasis [81]. Curcumin enhances the effectiveness of chemotherapy and radiotherapy, improves survival time, and reduces side effects, leading to better patient outcomes and quality of life. It inhibits tumor growth through mechanisms like reducing oxidative stress, suppressing inflammation, and inducing apoptosis. Curcumin’s low toxicity and multiple therapeutic benefits make it a promising supplement in cancer treatment [82]. Future research should focus on optimizing dosages, improving bioavailability, and further exploring its therapeutic potential.

Adriamycin is a chemotherapeutic agent widely used in the treatment of a variety of cancers. Its mechanism of action is multifaceted, targeting cancer cells through several pathways to inhibit their growth and proliferation. The primary mechanisms through which Adriamycin exerts its antitumor effects include its intercalation into DNA, inhibition of topoisomerase II, generation of free radicals, and induction of apoptosis [83]. Adriamycin acts by intercalating between base pairs in the DNA double helix. This intercalation disrupts the normal function of DNA by interfering with the processes of DNA replication and transcription. As a result, the cell’s ability to synthesize DNA and RNA is hindered, leading to the inhibition of cancer cell growth and multiplication. Adriamycin was demonstrated to inhibit topoisomerase II, an essential enzyme that modulates the topological states of DNA during replication and which can undergo redox cycling in cells to produce free radicals, particularly reactive oxygen species (ROS). Free radicals damage cellular components, including lipids, proteins, and nucleic acids. The oxidative stress resulting from this damage leads to cell death, particularly in cancer cells, which often have less effective antioxidant defenses than do normal cells. Adriamycin, recognized for its efficacy in combatting a number of solid tumors within clinical settings, encounters limitations due to its systemic toxicities, predominantly cardiotoxicity, and its susceptibility to the phenomenon of multidrug resistance [84]. The development of conjugated ADM complexes acting through glucose transporter 1 (GLUT1) and other receptors demonstrated potential to significantly reduce the adverse effects of systemic toxicity associated with traditional chemotherapy agents but also offers a novel strategy to overcome the formidable obstacle of multidrug resistance.

The identification of camptothecin, a pentacyclic quinoline alkaloid, marked a significant milestone in cancer research due to its demonstrated potential in exhibiting high cytotoxic activity across a diverse range of cell lines, indicating promising anticancer effects. Despite its potential, the clinical application of camptothecin faced substantial hurdles. Its poor solubility and unpredictable side effects presented significant challenges, ultimately preventing the drug from receiving approval for widespread use in oncological therapeutics. Camptothecin exists in a delicate equilibrium between an active lactone form and an inactive hydrolyzed carboxylate form. It is the active lactone form that is of particular interest, as it exhibits the ability to bind to the DNA topoisomerase I cleavage complex. This interaction is critical and is believed to represent the singular site of activity for camptothecin. By inhibiting the religation of DNA, camptothecin effectively triggers a cascade leading to apoptosis, or programmed cell death, a mechanism of paramount importance in the fight against cancer. In response to the limitations presented by camptothecin’s solubility and side effects, the scientific community has been motivated to develop a series of small molecule derivatives of camptothecin. These derivatives are designed with the intent to enhance the compound’s solubility, increase the stability of its lactone form, and improve overall bioavailability [85]. While these efforts have achieved varying levels of success, they represent a dedicated attempt to use the therapeutic potential of camptothecin more effectively. Moreover, the exploration into macromolecular agents has opened new frontiers in the optimization of camptothecin’s delivery and efficacy [86]. The modification of these agents, which include camptothecin molecules, either covalently appended or noncovalently associated with larger molecular structures, aims to address the drug’s limitations by improving its solubility and lactone stability.

Cannabidiol (CBD), both as a solitary agent and in conjunction with other compounds, has been demonstrated to effectively induce apoptosis in cancer cells; inhibit their migration and invasion in vitro; reduce tumor dimensions, vascularization, growth, and mass; and enhance survival rates, as well as provoke tumor regression in vivo [87]. The anti-proliferative effects of CBD have been specifically documented in cases of glioblastoma, indicating that apoptosis is triggered independently of cannabinoid receptors 1 and 2 (CB1 and CB2) and transient receptor potential vanilloid 1 (TRPV1) and vanilloid 2 (TRPV2). The synergistic application of CBD, γ-irradiation, and the ATM inhibitor KU60019 has been observed to elevate the expression of TNF/TNFR1 and TRAIL/TRAIL-R2 signaling, alongside death receptor 5 (DR5), within the extrinsic apoptotic pathway [88]. Furthermore, CBD has been found to activate the JNK-AP1 and NF-κB pathways, culminating in cell death. Unfortunately, the contributions of autophagy and cell cycle arrest to the CBD-mediated effects on glial cells have received comparatively limited attention.

Over the past century, significant advancements in the fields of medicinal plant biotechnology and microbiology have culminated in the development of several anticancer phytomedicines. The modern pharmacopeia includes a substantial number of remedies based on herbal formulations, encompassing clinically utilized anticancer drugs. Examples of these include drugs derived from podophyllotoxin, vinca alkaloids, curcumin, mistletoe plant extracts, taxanes, camptothecin, combretastatin, colchicine, artesunate, homoharringtonine, ellipticine, roscovitine, maytansine, tapsigargin, and bruceantin. Additionally, a variety of compounds such as psammaplin, didemnin, dolastatin, ecteinascidin, and halichondrin have been isolated from marine organisms, including microalgae, cyanobacteria, heterotrophic bacteria, and various invertebrates. These compounds have been evaluated for their anticancer activities in cellular and experimental animal models and in few instances, have been successfully incorporated into clinical chemotherapy regimens [89]. Natural products have historically been a crucial source of bioactive compounds, significantly advancing pharmaceutical discovery and development. Predominantly derived from microbes, fungi, and plants, these products have yielded many pharmacologically active agents, some of which exhibit anticancer properties. By enhancing cancer therapy through synergistic effects, natural products play a vital role in oncological treatment strategies. However, their natural origin typically excludes them from patent eligibility within the current legal framework [5,18].

### 6.4. Off-Patent and Repurposed Drugs

Despite the diverse mechanisms of action presented by unconventional compounds, the current patent system imposes significant limitations on their development as cancer therapeutics. An established exception is called drug repurposing, which permits the application of existing drugs in new therapeutic uses. Drug repurposing (or drug repositioning), involves identifying new indications for drugs already approved for other conditions. This approach substantially reduces the time and cost associated with drug development by utilizing existing data on safety, pharmacokinetics, and side effects. Drug repurposing can expedite the market introduction of treatments, providing new effective cancer therapeutic options. This method is especially valuable in oncology, offering a more rapid and cost-effective development process by leveraging existing data on drug safety, dosage, and toxicity (Figure 1).

Antidepressants, traditionally used for treating mood disorders, have shown potential anticancer effects, drawing attention to their possible repurposing for cancer therapy. The primary mechanisms behind anticancer action include the effects on cancer cell proliferation, apoptosis, and metastasis by altering serotonin receptor-mediated pathways, particularly through the 5-HT1A and 5-HT2A/2C receptors. The SSRIs and tricyclic antidepressants (TCAs) were shown to promote cancer cell apoptosis by regulating apoptotic factors, thereby activating caspase-dependent pathways and enhancing the expression of the tumor suppressor gene p53. Some compounds were demonstrated to suppress the formation of new blood vessels essential for tumor growth by reducing vascular endothelial growth factor (VEGF) expression and influencing other angiogenic factors. Antidepressants may also affect the immune response, potentially inhibiting tumor growth by altering natural killer cell activity, enhancing phagocytosis, and modifying cytokine secretion [90]. Bupropion, a medication primarily known for its application in treating major depressive disorder and as an aid in smoking cessation, was demonstrated to have certain anticancer properties. Although not conventionally classified within the spectrum of anticancer agents, emerging research suggests that bupropion may exhibit anticancer activity through inducing cell cycle arrest and apoptosis. The pro-apoptotic effects were shown to be mediated through the BCL-2 family proteins, which play pivotal roles in the intrinsic pathway of apoptosis. The varied mechanisms of antidepressants, ranging from modulating serotonin signaling to impacting cancer cell metabolism, offer a promising avenue for cancer treatment [91]. Further research is essential to validate their efficacy and safety in oncology.

Verapamil, traditionally known for its role as a calcium channel blocker in the management of cardiovascular diseases, demonstrated anticancer effects involving the modulation of calcium homeostasis, alteration of cell cycle progression, and interference with multidrug resistance (MDR) pathways. Verapamil inhibits voltage-gated calcium channels (VGCCs) on the cell membrane and disrupts the calcium-dependent signaling pathways critical for tumor cell growth and survival, thereby exerting an antiproliferative effect on cancer cells. It can also induce cell cycle arrest, particularly at the G0/G1 phase, leading to the inhibition of cancer cell proliferation [92]. The mechanism involves the modulation of cyclin-dependent kinases (CDKs) and their regulatory cyclins, which are essential for cell cycle progression. One of the major challenges in cancer chemotherapy is the development of multidrug resistance (MDR), in which cancer cells become resistant to a wide range of anticancer drugs. Verapamil has been identified as an inhibitor of the P-glycoprotein (P-gp) efflux pump, a key player in the MDR phenomenon. By inhibiting P-gp, verapamil can increase the intracellular accumulation of chemotherapeutic drugs, enhancing their cytotoxicity and overcoming drug resistance. The induction of apoptosis was demonstrated to occur through both intrinsic and extrinsic pathways. While verapamil has shown potential in augmenting anticancer therapies, its therapeutic application must be approached with caution due to the associated toxicity risks. Its toxicity, particularly at the higher doses required for anticancer effects, poses significant challenges [93].

Ivermectin, initially developed as an antiparasitic agent, exhibits potential anticancer properties through multiple mechanisms. These include the inhibition of the WNT/TCF signaling pathway, which is often dysregulated in cancerous cells, leading to uncontrolled proliferation. Additionally, ivermectin induces apoptosis in cancer cells by activating cell death receptors and disrupting mitochondrial function, thereby initiating programmed cell death. It also inhibits PAK1 activity, a kinase frequently overexpressed in malignancies, contributing to tumorigenesis [94]. Ivermectin possesses anti-inflammatory properties that mitigate the chronic inflammation associated with cancer progression. It impedes cancer cell migration and invasion by regulating proteins and enzymes involved in extracellular matrix remodeling and cell adhesion. Ivermectin disrupts autophagy, a survival mechanism that cancer cells exploit under stress, including chemotherapy, thus reducing their proliferation. Furthermore, it targets cancer stem cells, a subpopulation responsible for tumor initiation, metastasis, and resistance to conventional therapies [95]. Given these multifaceted mechanisms, ivermectin presents as a promising candidate for anticancer therapy; however, additional research and clinical trials are necessary to establish optimal dosing and a safety profile in the oncological setting.

The anticancer efficacy of thalidomide and its derivatives, known as immunomodulatory drugs (IMiDs), is attributed to their capacity to modulate the immune system, inhibit angiogenesis, directly induce tumor cell apoptosis, reduce pro-inflammatory cytokine production, and alter cell adhesion molecule expression. Thalidomide enhances the immune response against malignancies by increasing the production of interleukin-2 (IL-2) and interferon gamma (IFN-γ). This augmentation of IL-2 and IFN-γ levels facilitates the activation of T cells and natural killer (NK) cells, thereby bolstering the immune-mediated eradication of tumor cells [96]. The drug impedes the neovascularization essential for tumor growth and dissemination by downregulating vascular endothelial growth factor (VEGF) and basic fibroblast growth factor (bFGF), crucial regulators of angiogenesis. Also, through the cereblon pathway, thalidomide induces the degradation of specific transcription factors vital for cancer cell survival, leading to the apoptosis of cancer cells. Several studies demonstrated that the modulation of adhesion molecule expression on the surface of tumor cells by thalidomide affects their adhesive, invasive, and metastatic potential, further contributing to its anticancer activity. Thalidomide and its derivatives are employed in the treatment of multiple myeloma and certain leukemias and lymphomas [97]. Ongoing research aims to elucidate the intricate mechanisms through which thalidomide exerts its effects, with the goal of optimizing therapeutic outcomes and minimizing adverse effects.

Sildenafil, primarily recognized for its efficacy in treating erectile dysfunction, has exhibited potential anticancer properties that involves the inhibition of PDE-5, leading to elevated cyclic guanosine monophosphate (cGMP) levels within tumor cells. Sildenafil results in increased caspase-3 levels; reduced NF-κB, BCL-2, cyclin D1, ICAM-1, and MMP-2 levels; normalized Nrf2; and notably improved histological patterns [98]. Elevated cGMP levels activate protein kinase G (PKG), thereby inhibiting cell proliferation, inducing apoptosis, and mitigating cancer cell migration and metastasis. Sildenafil was shown to modulate the tumor microenvironment, enhancing chemotherapeutic drug delivery and impeding angiogenesis by downregulating pro-angiogenic factors. It may augment anticancer immune responses by enhancing tumor immunogenicity and reducing immunosuppressive conditions within the tumor microenvironment. Sildenafil has also been noted to directly induce apoptosis and senescence in various cancer cell types, an effect partly mediated through the induction of reactive oxygen species (ROS) and the disruption of mitochondrial integrity [99]. Despite these promising findings, derived predominantly from preclinical research studies, clinical trials are imperative to ascertain sildenafil’s safety, efficacy, and optimal application as an anticancer agent.

Propranolol, traditionally used to treat cardiovascular conditions, has shown potential anticancer properties through various mechanisms. Its anticancer effects include inhibiting cancer cell proliferation by blocking β-adrenergic receptors, which in turn reduces epidermal growth factor receptor (EGFR) transactivation critical for cancer cell growth. It also induces apoptosis in cancer cells by activating intrinsic apoptotic pathways, leading to cell death. Additionally, propranolol possesses anti-angiogenic effects, hindering the formation of new blood vessels essential for tumor growth and metastasis, by reducing the production of vascular endothelial growth factor (VEGF) and hypoxia-inducible factor-1α (HIF-1α). It suppresses metastasis by decreasing matrix metalloproteinases (MMPs) and inhibiting adrenergic signaling pathways involved in cell migration and invasion [100]. Emerging evidence suggests that propranolol may also modulate the immune response, enhancing the activity of immune cells against cancer cells. These multifaceted mechanisms underscore the potential of repurposing propranolol as an adjunctive therapy in cancer treatment, requiring further research to fully exploit its therapeutic capabilities [101].

Digoxin, traditionally used to treat heart conditions, has shown potential anticancer effects through multiple molecular mechanisms. Its primary action, the inhibition of the Na^+^/K^+^-ATPase pump, disrupts the cellular ion balance, leading to increased intracellular calcium. This action triggers signaling pathways activating calcineurin and NFAT, which inhibit cancer cell growth, induce apoptosis, and can potentially reduce metastasis. These diverse mechanisms highlight digoxin’s potential for cancer therapy, suggesting further investigation for clinical applications. Digoxin impacts the hypoxia-inducible factor-1α (HIF-1α) pathway, inhibiting tumor growth under hypoxic conditions common in the tumor microenvironment. Additionally, it interferes with the AKT/mTOR pathway, essential for cell proliferation, and modulates BCL-2 family proteins to promote apoptosis in cancer cells [102]. These mechanisms highlight digoxin’s potential as an anticancer agent, suggesting further research regarding its clinical application in oncology. One study found that digoxin offers no advantage over β-blockers in regards to cancer prevention. β-blockers show potential as a new candidate for cancer prevention, warranting further clinical evaluation. The study also highlights the importance of competing risk analysis in similar clinical research [103].

Tamoxifen, a selective estrogen receptor modulator (SERM), plays a pivotal role in the management of estrogen receptor (ER)-positive breast cancer due to its multifaceted mechanism of action that exerts anti-estrogenic effects on breast tissue. Tamoxifen decreases the expression of cyclin D1 and increases the expression of p21, leading to cell cycle arrest in the G1 phase. It interferes with the signaling pathways of growth factors such as EGFR and IGF-1, which are involved in cell proliferation and survival [104]. The effectiveness in reducing the risk of breast cancer recurrence and mortality has been well documented, making it a cornerstone in the adjuvant therapy for ER-positive breast cancer patients. The anticancer mechanism of action of tamoxifen is primarily attributed to its ability to competitively bind to estrogen receptors on breast cancer cells. Tamoxifen blocks the estrogen–ER interaction that is crucial for the proliferation and survival of cancer cells. Tamoxifen has been shown to induce changes in the expression of genes that regulate cell cycle progression and apoptosis, further contributing to its anticancer effects. Tamoxifen treatment has been associated with an increased expression of pro-apoptotic genes and a decreased expression of genes that promote cell survival and proliferation. This modulation of gene expression facilitates the arrest of cancer cells in the G0/G1 phase of the cell cycle and promotes apoptotic cell death, reducing the growth of estrogen-dependent breast cancer cells. Also, several studies demonstrated that tamoxifen exerts its anticancer effects through modulation of the tumor microenvironment, influencing the secretion of various growth factors and cytokines by cancer cells and stromal cells, which can affect tumor growth, angiogenesis, and metastasis. By altering the tumor microenvironment, tamoxifen not only inhibits the direct growth of cancer cells but also impedes the processes that support tumor progression and dissemination [105]. These mechanisms collectively contribute to the inhibition of the growth and proliferation of ER-positive breast cancer cells, underscoring the importance of tamoxifen in the management of breast cancer.

Salinomycin is a well-known polyether antibiotic that has been identified as a selective inhibitor of breast cancer stem cells. Salinomycin was found to selectively kill cancer stem cells (CSCs) in various types of cancers, including breast, lung, and colorectal cancers. The Wnt/β-catenin signaling pathway plays a crucial role in the regulation of CSCs and is implicated in the development and progression of various cancers. Salinomycin has been shown to inhibit the Wnt/β-catenin pathway, thereby inhibiting the growth and proliferation of CSCs. Salinomycin disrupts the mitochondrial membrane potential, leading to the release of cytochrome c and activation of the intrinsic pathway of apoptosis. The compound was shown to act as an ionophore, facilitating the transport of ions across cellular membranes; it specifically disrupts potassium and sodium ion gradients in cancer cells. Some studies suggest that salinomycin can induce autophagy, a cellular process involved in the degradation and recycling of cellular components. While autophagy can promote cell survival under stress conditions, excessive or dysregulated autophagy can lead to cell death, and salinomycin-induced autophagy may contribute to its anti-cancer effects. Also, the compound was demonstrated to inhibit the epithelial-to-mesenchymal transition (EMT), thereby potentially limiting the metastatic potential of cancer cells. The mechanism of action of salinomycin underscores its potential as a therapeutic agent against cancer, particularly in targeting CSCs and overcoming drug resistance [106,107]. However, further research and clinical trials are needed to fully understand its efficacy and safety profile in cancer therapy.

Metformin, a biguanide commonly used to treat type 2 diabetes mellitus, has been shown to activate AMP-activated protein kinase (AMPK), a key energy sensor in cells that maintains energy balance via the inhibition of the mammalian target of rapamycin (mTOR). Additionally, it inhibits the Warburg effect by acting on complex I of the mitochondrial electron transport chain and reduces ATP production, forcing cells to rely more on oxidative phosphorylation, which can be a less favorable condition for rapidly growing cancer cells [108]. Metformin reduces insulin resistance and lowers circulating insulin levels; however, since insulin and IGF can promote tumor growth by activating insulin and IGF receptors on cancer cells, lowering these levels with metformin also reduces tumor growth and proliferation. Beyond its systemic effects, metformin has been shown to exert direct effects on cancer cells, including the induction of cell cycle arrest and apoptosis. Recent studies suggest that the compound may also affect the tumor microenvironment by reducing inflammation and oxidative stress within the tumor microenvironment, potentially limiting tumor growth and metastasis. Metformin was shown to have an impact on cancer stem cells. The potential anti-cancer effects of metformin are supported by epidemiological studies showing reduced cancer incidence and mortality among diabetic patients taking metformin. However, while preclinical and some clinical studies suggest the promising anti-cancer effects of metformin, more research and clinical trials are needed to fully understand its efficacy and mechanism of action in cancer therapy [109].

Disulfiram, traditionally used to treat chronic alcoholism by producing sensitivity to alcohol, has been shown to inhibit proteasome, a complex that degrades unneeded or damaged proteins, leading to the accumulation of proteins that induce cell cycle arrest and apoptosis, thereby inhibiting cancer cell growth and survival [110]. Disulfiram can induce oxidative stress in cancer cells by generating elevated levels of reactive oxygen species (ROS) that damage cellular components, leading to cell death. Disulfiram is a chelator of copper and can form a complex with copper (Cu) in the bloodstream that inhibits metalloenzymes essential for tumor growth and survival (e.g., SOD1 and NF-κB pathways). Disulfiram is an inhibitor of ALDH, an enzyme involved in alcohol metabolism and also expressed in some cancer cells, particularly in CSCs. Similar to other cancer therapies that target cancer stem cells (CSCs), disulfiram has been found to affect CSCs in various types of cancers. The multi-targeted nature of disulfiram’s action against cancer cells, along with its ability to selectively target cancer cells and CSCs, make it a promising candidate for cancer therapy [111].

### 6.5. Small Molecules

Dimethyl sulfoxide (DMSO) is an organic solvent known for its diverse biological activities, including its potential anticancer properties. DMSO has demonstrated potential anticancer properties through various mechanisms, including apoptosis, cell cycle arrest, and promoting differentiation in cancer cells. DMSO also increases reactive oxygen species (ROS) production, leading to oxidative stress and cell death [112]. Additionally, it modulates gene expression and enhances the efficacy of certain chemotherapeutic agents [113]. Despite promising in vitro and in vivo results, the clinical use of DMSO is limited by its side effects and toxicity at higher doses. Moreover, DMSO, widely used due to its solvent properties and low toxicity at concentrations below 10%, was reported to induce retinal apoptosis in vivo at low concentrations, raising safety concerns for its use in biological assays and drug solubilization, with recommendations for the employment of alternative solvents or strict controls when DMSO must be used [114]. Further research is needed to optimize its therapeutic applications in oncology.

Vitamin C, also known as ascorbic acid, has been studied for its potential role in cancer treatment. Reports have demonstrated that ascorbic acid exerts both direct and indirect effects on cancer cells and the tumor microenvironment. Vitamin C can act as a pro-oxidant or an antioxidant, depending on pharmacological concentration. Vitamin C can inhibit HIF-1, a transcription factor that promotes cancer cell survival under low oxygen conditions, and stabilize prolyl hydroxylase, an enzyme that degrades HIF-1. Vitamin C also reduces the adaptation of cancer cells to hypoxic conditions, impairing their growth and survival. Reports have demonstrated that vitamin C promotes collagen production and strengthens the extracellular matrix, which may hinder tumor invasion and metastasis. By enhancing immune functions, including the activity of natural killer cells, T-lymphocytes, and the production of interferon, it helps the immune system to target and destroy cancer cells more effectively. Vitamin C is also involved in the regulation of DNA and histone demethylation, serving as a cofactor for ten-eleven translocation (TET) enzymes, which are involved in DNA demethylation [115]. This can restore the expression of tumor suppressor genes that are often silenced in cancer cells through hypermethylation. The antioxidative and anti-inflammatory properties of ascorbic acid are known to reduce the production of pro-inflammatory cytokines and inhibit the NF-κB pathway, which is often activated in cancer cells [116]. High-dose vitamin C has been shown to disrupt glycolysis in cancer cells by depleting the cofactor NAD+, leading to an energy crisis and cancer cell death via apoptosis [117]. Clinical trials have also been conducted to assess the efficacy of high-dose vitamin C in cancer patients, both as a monotherapy and in combination with conventional treatments like chemotherapy and radiation. Some studies have shown promising results, but the effectiveness can vary depending on the type and stage of cancer, dosage, and method of administration. While the potential anti-cancer mechanisms of vitamin C are supported by preclinical studies, further research and well-designed clinical trials are needed to establish its efficacy and optimal use in cancer therapy [118,119]. The combination of vitamin C with other treatments may offer synergistic benefits, but this should be approached with careful consideration of patient-specific factors and under medical supervision.

Dichloroacetate (DCA) has a variety of industrial and medical applications, and it is known for affecting cell metabolism. DCA was shown to activate the pyruvate dehydrogenase complex (PDC), a critical enzyme complex in the mitochondrial matrix that catalyzes the conversion of pyruvate to acetyl-CoA, a step in cellular energy production, via the citric acid cycle. Cancer cells typically exhibit altered metabolism, notably the Warburg effect, in which they rely heavily on glycolysis for energy production, even in the presence of oxygen. DCA was demonstrated to target this metabolic pathway by inhibiting pyruvate dehydrogenase kinase (PDK), which in turn activates PDC, forcing cancer cells to revert to oxidative phosphorylation for energy production. This shift leads to the increased production of reactive oxygen species within cancer cells, promoting apoptosis and associated tumor growth inhibition. DCA has been shown to have a synergistic effect with certain chemotherapeutic agents, enhancing their efficacy against cancer cells by sensitizing them to drug-induced apoptosis. Anti-cancer activity was demonstrated across a variety of cancer models, including breast cancer, leukemia, colorectal cancer, glioblastoma, liver cancer, and several others [120,121]. While DCA has shown anticancer activity, its therapeutic use is limited by side effects such as peripheral neuropathy and potential carcinogenicity [122]; therefore, the research focus has shifted toward combining DCA with other treatments and the development of DCA analogs to increase its effectiveness while minimizing toxicity. Research regarding the DCA analogs [123] systematically evaluated eight analog molecules, each characterized by a conserved dichloric terminus, for their potential anticancer efficacy both in vitro and in vivo. Among these, two compounds (DCAH and DMAH) demonstrated U87 glioblastoma tumor growth inhibition in vivo, thereby underscoring their potential clinical utility as accessible chemotherapeutic agents, after their safety profiles are studied and evaluated.

The in vivo effectiveness of DCAH and DCMAH against difficult to treat glioblastoma demonstrated their potential as more effective and potentially less toxic therapeutic options. Through collaborative efforts, researchers at Altogen Labs (Austin, TX, USA) and the National Cancer Institute (Bethesda, MD, USA) found that DCAH and DCMAH operated differently from DCA, as evidenced by varying levels of key proteins, indicating that these compounds may represent a new class of chemicals with potential chemotherapeutic applications [123]. These compounds demonstrated profound inhibition of tumor growth in the U87 glioblastoma xenograft model, indicating their potential utility as clinically relevant anticancer agents. Despite the promising outcomes of the study, both DCAH and DCMAH are unpatentable within the current patent system framework due to prior public disclosure. As unpatentable entities, securing research funding for further development and clinical trials has been challenging. Since the publication of these findings in 2019, the absence of funding has halted research progress, leaving no ongoing efforts to advance the clinical application of DCAH and DCMAH. This scenario underscores a common barrier in drug development, in which promising compounds may not reach clinical application due to financial and regulatory hurdles, particularly when patent protection cannot be secured to justify the investment in costly preclinical studies and follow-up clinical trials. This example highlights a prevalent challenge in the realm of drug development, in which the inability to obtain patent protection can deter the advancement of promising therapeutic agents, primarily due to the financial and regulatory commitments required for comprehensive clinical trials.

## 7. Challenges and Future Directions

One of the major challenges in the development of new cancer treatments involves the regulatory approval process. Unconventional compounds, especially those that are natural or not patent-protected (Table 4), do not attract investments from pharmaceutical companies for conducting preclinical testing of efficacy and safely or rigorous clinical trials. The current patenting system framework results in very limited preclinical research funding options, leaving the safety and efficacy of many unconventional compounds unknown to the public. Preclinical research is necessary to establish the mechanisms of action and efficacy of such compounds. Understanding the precise mechanisms through which the compound acts against cancer cells is crucial. The complexity of cancer biology means that demonstrating clear, effective, and reproducible anticancer activity in clinical settings can be challenging. Ensuring the consistent quality, potency, and purity of unconventional compounds can be difficult. Natural products, for example, can vary significantly depending on the source, extraction method, and storage conditions. Like any therapeutic agent, unconventional compounds must be thoroughly evaluated for safety. Natural does not always mean safe, and some compounds may have toxic effects or harmful interactions with other medications. The lack of patent protection can deter investment in the necessary research and development to bring a compound to market. Finding a model that allows for the recovery of these investments, while keeping the treatments accessible and affordable, is a challenge.

Patent law, meant to encourage innovation, can sometimes lead to “negative innovation”, where patents incentivize harmful practices rather than benefiting patients. Negative innovation occurs when patents drive the development of products that are not only suboptimal but harmful to consumers. The case of ibrutinib, a cancer drug, illustrates this issue. Despite evidence showing that lower doses of ibrutinib could be effective with fewer side effects, the patent system incentivized the company to push higher, more toxic doses to maintain patent protection [124]. Addressing the challenge of negative innovation involved in patents, particularly in regards to pharmaceuticals, necessitates targeted strategies due to the overlap between patents fostering negative and positive innovation. It is important to implement a stringent utility criterion in patent law that could differentiate innovations that genuinely contribute to social welfare. This involves a certification of potential benefits at the patent application stage, followed by empirical verification, to maintain patent validity. Enhancing postmarketing surveillance and mandating Phase 4 clinical trials would further ensure real-world efficacy and cost-effectiveness. Also, enhancing communication between regulatory agencies could reduce inconsistencies and exploitation in the innovation process, supporting socially beneficial innovations. Finally, streamlining and capping the various forms of pharmaceutical product exclusivity could deter strategic manipulation of the patent system, promoting a more equitable innovation landscape [124]. These strategies aim to curb negative innovation by prioritizing social welfare, fostering regulatory coherence, and moderating exclusivity rights.

Regulatory bodies can adapt current frameworks to better accommodate the development and approval of unconventional cancer therapies. This could include creating new pathways for the approval of natural products or repurposed drugs. The patent system, originally crafted to foster innovation within the realms of pharmaceuticals and medical devices, has paradoxically been a source of controversy. Critics argue that it has led to escalated healthcare costs and obstructed further research by imposing restrictions on the availability of patented materials. A pertinent illustration of this is the U.S. Orphan Drug Act. While it has undeniably accelerated the development of novel treatments for rare cancers, many of which have later been adapted for broader applications in more prevalent diseases [125], the initiative highlights a complex landscape. The oncology sector, in particular, has been a notable beneficiary and contributor to orphan drug research, a testament to which is the fact that over one-third of all orphan drugs have been approved by the US FDA. Despite these advancements, the commendation of the US FDA’s efforts in promoting orphan drug development underscores a broader issue: the measures, though impactful, are not comprehensive enough to address the entirety of the challenges facing the healthcare system. The reliance on patents as a primary incentive for innovation does foster a degree of progress, yet it also creates barriers that can stifle broader research endeavors and inflate healthcare costs, thereby limiting the overall accessibility of essential medical treatments.

Collaborative models developed between academic institutions, government, and private entities could help bridge the funding gap for necessary preclinical and clinical studies of unconventional anticancer compounds. Crowdsourcing and open-source drug discovery platforms are novel approaches to provide currently lacking funding for unpatentable anticancer compounds. Utilizing high-throughput screening may accelerate the identification of potential anticancer compounds from vast libraries of unconventional molecules. Also, genetic profiling of tumors can help identify patient subgroups that are more likely to respond to certain unconventional compounds, optimizing treatment efficacy and reducing side effects.

Crowdfunding has democratized research funding, enabling support for projects, particularly in less common or emerging areas, that may not attract traditional funding [126]. By directly engaging the public, crowdfunding has provided a vital funding stream for these underfunded areas, rare cancers, and personalized medicine, thereby accelerating the research and development of new therapies. As personalized approaches become central to cancer therapy, the ability to fund these specialized research projects through public contributions could significantly enhance treatment outcomes [127]. Despite its benefits, crowdfunding presents challenges, particularly in regards to ensuring the accuracy of information provided to donors and in managing public expectations [128]. The success of crowdfunding in cancer research depends on addressing these challenges, while leveraging its potential to fund innovative and high-risk research. Overall, crowdfunding has expanded the financial resources available for cancer research, offering a promising avenue for advancing the field, particularly in areas that are traditionally underfunded or require rapid, flexible support.

Artificial intelligence (AI) is revolutionizing drug discovery and development by expediting complex processes. Advances in AI and machine learning offer transformative opportunities for identifying disease targets, predicting drug interactions, and optimizing drug formulations. AI algorithms analyze vast amounts of biological data, such as genomic and proteomic information, to enhance targeted drug discovery and increase successful approvals. AI also reduces development costs by optimizing research processes and predicting pharmacokinetics and toxicity, minimizing the need for extensive animal testing. Additionally, AI facilitates personalized medicine by analyzing patient data for more effective treatments and improved adherence. AI’s development has resulted in applications for drug discovery, dosage form design, process optimization, testing, and pharmacokinetics/pharmacodynamics (PK/PD) studies [129,130]. The application of AI in cancer research has garnered significant attention due to its potential to revolutionize various aspects of oncology. AI technologies, particularly machine learning and deep learning, are being increasingly integrated into cancer diagnosis, treatment, and management, offering promising advancements in accuracy, efficiency, and personalized care. Diagnostic applications of AI have shown exceptional capabilities in enhancing the diagnostic accuracy of various cancers through imaging technologies. For instance, a recent study demonstrated the effectiveness of AI in recognizing breast cancer from fine-needle aspirated tissue samples by utilizing nuclear features, which significantly improves early detection and diagnosis [131]. Similarly, AI-aided imaging systems were demonstrated to enhance pulmonary nodule detection, which is crucial for early-stage lung cancer diagnosis [132]. The integration of AI into the identification of treatment-resistant cancer cells has been used with machine learning to unveil distinct RNA methylation regulators in pan-cancer neoadjuvant therapy, potentially leading to more effective personalized treatment plans [133]. Additionally, AI was recently used to predict the completion of chemotherapy in pancreatic cancer patients, highlighting its utility in improving patient adherence to treatment protocols [134]. AI-driven predictive models and risk assessment are being developed to forecast and prevent chronic diseases, including various forms of cancer, by analyzing vast datasets of patient information. This proactive approach is pivotal in identifying at-risk individuals and implementing early interventions; however, there are inherent challenges and ethical considerations that must be addressed. A recent study emphasized the importance of not overlooking the limitations of AI, such as potential biases in data and the need for transparency in AI-driven decision-making processes [135]. These challenges must be carefully managed to ensure that AI tools are both effective and equitable in cancer care. The integration of AI in cancer research is undoubtedly a transformative force, offering unprecedented opportunities to improve diagnostic accuracy, personalize treatment, and enhance predictive analytics. However, the successful implementation of AI technologies in clinical settings requires careful consideration of ethical implications, continuous validation, and collaboration between AI experts and healthcare professionals.

Cancer is a global issue, and solutions can come from any corner of the world. International collaborations can help in sharing knowledge, resources, and compounds of interest, potentially speeding up the discovery and development process. Educating both the medical community and the public about the potential and limitations of unconventional cancer therapies is crucial. Advocacy can also play a role in pushing for more research and better regulatory pathways. Research and development in this area hold the promise of uncovering cost-effective, accessible cancer treatments that could complement or offer alternatives to current therapies. The journey from discovery to clinical application is complex and fraught with challenges, but with concerted effort and innovative approaches, significant strides can be made in improving cancer care.

## 8. Conclusions

This paper presents a comprehensive review of alternative cancer therapeutics, emphasizing their healing potential and the challenges they face in clinical application. Unconventional anticancer compounds, including off-patent drugs, natural products, and repurposed medications, offer promising and cost-effective options for enhancing cancer treatment. These compounds act through diverse mechanisms, such as targeting specific molecular pathways and cancer hallmarks and improving the efficacy of existing therapies. However, their development is significantly hindered by the prevailing patent system and its associated financial models.

The current structure of intellectual property protection in drug development inherently favors the modification of existing molecules to create patentable derivatives rather than the exploration of non-patentable, yet potentially effective and safe, alternatives. This bias in research funding towards patentable compounds constrains the diversity of chemical molecules investigated for cancer therapy and narrows the scope of cancer research. The lack of financial incentives for pharmaceutical companies to invest in preclinical and clinical trials for non-patentable compounds further limits their availability in standard treatment protocols.

To address this critical gap, there is a pressing need for a shift in patenting system criteria and funding priorities. Allocating adequate grant funding for the investigation of non-patentable molecules would disrupt the current paradigm, enabling research into a wider array of substances that fall outside of the patent protection system, yet hold significant potential to advance cancer treatment. Moreover, regulatory reforms are essential to facilitate the development and approval of these unconventional therapies, ensuring that their potential benefits can be fully realized in clinical practice.

In conclusion, while unconventional cancer therapeutics hold great promise, their advancement requires significant changes in the patenting system, funding structures, and regulatory frameworks. By addressing these challenges, the field of oncology can expand its therapeutic options, offering new hope to cancer patients globally.

## Figures and Tables

**Table 2:** Potential modifications to patent criteria for alternative cancer therapeutics.

Expanded Definitions of Novelty and Non-Obviousness	Allow incremental innovations and broaden non-obviousness for significant clinical benefits.
Patent Eligibility for Naturally Derived Substances	Allow patents for novel formulations and combinations with conventional drugs.
Reduced Stringency for Early-Stage Innovations	Allow provisional patents for early-stage therapies, with a fast-track for rare cancers.
Incentivizing Research on Synergistic Combinations	Allow less stringent patenting criteria for multi-modal approaches and synergistic combinations
Data-Driven Patents for Personalized Medicine	Grant patents for precision medicine and AI-driven combination therapies.
Collaboration and Shared Patents	Encourage co-patent models and patent pools for collaborative development.
Incentives for Open Innovation and Public Health Benefits	Extend patent periods for unmet needs and offer tax credits for socially responsible licensing.

**Table 4:** Spectrum of different cancer suppression mechanisms of action by selected adjuvant chemotherapy compounds.

Compound	Mechanism of Action
Adriamycin (e.g., Doxorubicin)	This drug intercalates into the DNA, disrupting the function of topoisomerase II, which leads to breaks in double-stranded DNA and prevents DNA and RNA synthesis. These drugs also generate free radicals, causing additional damage to cellular components. Adriamycin is used in the treatment of a wide variety of cancers, including breast cancer, lymphomas, and leukemia.
Platinating agents (e.g., Cisplatin, Carboplatin)	These are chemotherapy drugs that work by forming platinum–DNA adducts, which in turn interfere with DNA replication and transcription. This leads to cell cycle arrest and apoptosis. Platinates are used to treat various cancers, including lung, ovarian, and testicular cancer.
Camptothecins (e.g., Irinotecan, Topotecan)	These drugs inhibit the enzyme topoisomerase I, which is essential for DNA replication. By stabilizing the temporary breaks that topoisomerase I creates in the DNA double helix, camptothecins cause DNA damage that leads to cell death. Camptothecins are used in the treatment of colorectal, ovarian, and small cell lung cancer, among other conditions.
Salicylic Acid (e.g., Aspirin)	Salicylic acid inhibits the activity of cyclooxygenase enzymes (COX-1 and COX-2), leading to a reduction in the synthesis of prostaglandins, which are involved in inflammation and may play a role in cancer progression. It works through activation of the AKT/mTOR and AMPK-dependent pathways and is associated with a reduced risk of colorectal cancer.
Statins (e.g., Simvastatin, Pitavastatin)	Statins affect RAS and Rho isoprenylation, signal transduction, and DNA synthesis. Statins regulate autophagy, which plays a crucial role in the tumor suppressive process. Statins can induce ferroptosis and pyroptosis.
Antidepressants (e.g., Bupropion, Duloxetine)	These drugs impact cancer cell growth, death, and spread by affecting serotonin pathways, especially through the 5-HT1A and 5-HT2A/2C receptors. SSRIs and TCAs encourage cancer cell death by influencing apoptotic pathways and boosting the tumor suppressor gene p53. They reduce VEGF expression, potentially slowing tumor growth by affecting natural killer cells and enhancing phagocytosis.
Verapamil	A calcium channel blocker, verapamil has demonstrated potential anticancer effects through its interference with multidrug resistance (MDR) pathways and plays a functional role in cell cycle alteration.
Curcumin	The active component of turmeric, curcumin exhibits anti-inflammatory and anticancer properties, likely through the modulation of various molecular targets, including NF-κB, COX-2, and p53. Clinical utility has been limited by poor bioavailability, prompting research into formulation strategies.
Phytochemicals (e.g., Cannabidiol, CBD)	Phytocannabinoids from the cannabis plant exhibit a variety of proposed mechanisms, including anti-inflammatory, antioxidant, and anticonvulsant effects. CBD has been shown to induce apoptosis and inhibit cancer cell proliferation and angiogenesis via multiple cellular pathways.
Ivermectin	Ivermectin is a macrolide antiparasitic drug that is widely used for the treatment of many parasitic diseases. It suppresses cancer cell proliferation, cell cycle arrest, metastasis, and proliferation via inhibition of the PAK1 signaling and WNT/TCF pathway.
Thalidomide	Originally developed as a sedative, thalidomide has been repurposed to exhibit significant anticancer properties through a multifaceted mechanism of action, including cytokine action and modulating the release of inflammatory mediators like TNF-α.
Sildenafil	Sildenafil is a drug primarily prescribed for the treatment of erectile dysfunction. It exerts its biological effects through the inhibition of phosphodiesterase PDE-5.
Propranolol	Propranolol induces apoptosis and inhibits cancer cell proliferation by blocking the β-adrenergic receptors, thereby disrupting growth factor signaling. It exhibits anti-angiogenic properties by reducing factors essential for tumor blood vessel formation.
Digoxin	Digoxin, known for treating heart conditions, exhibits potential anticancer effects by inhibiting the Na^+^/K^+^-ATPase pump, affecting cellular ion balance, and increasing intracellular calcium. Digoxin targets the HIF-1α and AKT/mTOR pathways, essential in hypoxic tumor environments and cell proliferation.
Tamoxifen	A selective estrogen receptor modulator (SERM), tamoxifen is crucial in treating estrogen receptor-positive breast cancer due to its ability to block estrogen’s effects on breast tissue. Tamoxifen binds to estrogen receptors, preventing estrogen from promoting cancer cell growth. It modulates gene expression to inhibit cell proliferation and induce apoptosis, contributing to its anticancer effects.
Salinomycin	Identified as a potential cancer stem cell (CSC) targeting agent, salinomycin selectively kills CSCs over non-stem cancer cells, potentially offering a way to overcome resistance and prevent tumor recurrence.
Metformin	Originally developed as an antidiabetic drug, metformin has been observed to exhibit anticancer effects, likely through the activation of AMP-activated protein kinase (AMPK), which in turn inhibits the mTOR pathway, leading to decreased protein synthesis and cell proliferation.
Disulfiram	Used in the treatment of alcohol dependence, disulfiram has shown potential as an anticancer agent by inhibiting proteasome, leading to the accumulation of misfolded proteins and inducing cancer cell death. Disulfiram also likely chelates copper, forming a complex that induces oxidative stress and kills cancer cells.
Dichloroacetate	This drug modulates the activity of pyruvate dehydrogenase, an enzyme that influences cellular metabolism. By shifting cancer cell metabolism from anaerobic glycolysis to glucose oxidation, DCA promotes apoptosis in cancer cells, likely through the Warburg effect, a characteristic metabolic alteration observed in many cancer cells.

## Data Availability

The data are openly available in a public repository that issues datasets with DOIs.
